# Frequency of juvenile idiopathic arthritis and associated uveitis in pediatric rheumatology clinics in Turkey: A retrospective study, JUPITER

**DOI:** 10.1186/s12969-021-00613-2

**Published:** 2021-08-23

**Authors:** Sezgin Sahin, Ceyhun Acari, Hafize Emine Sonmez, Fatma Zehra Kilic, Erdal Sag, Hatice Adiguzel Dundar, Amra Adrovic, Selcan Demir, Kenan Barut, Yelda Bilginer, Betul Sozeri, Erbil Unsal, Seza Ozen, Ozgur Kasapcopur

**Affiliations:** 1 Istanbul University-Cerrahpasa, School of Medicine, Koca Mustafapaşa Cd. No:53, Fatih, 34098 Istanbul, Turkey; 2 Dokuz Eylul University, School of Medicine, Izmir, Turkey; 3 Hacettepe University, School of Medicine, Ankara, Turkey; 4 Umraniye Training and Research Hospital, Istanbul, Turkey

**Keywords:** Juvenile idiopathic arthritis, Frequency, Uveitis, HLA-B27, ANA, Turkey

## Abstract

**Background:**

Juvenile idiopathic arthritis (JIA), is the most common pediatric rheumatologic disorder with unknown etiology. Currently, no population-based data are available regarding the distribution of categories and frequency of uveitis in patients with JIA in Turkey. The purpose of this study was to evaluate the frequency of JIA-associated uveitis (JIAU) and distribution of JIA categories in a Turkish JIA cohort.

**Methods:**

This was a retrospective study of 500 randomized patients in four pediatric rheumatology clinics in Turkey.

**Results:**

Oligoarticular JIA (oJIA) was the most common JIA disease category in this study cohort (38.8%). The frequencies of the other categories were as follows: enthesitis-related arthritis (ERA), 23.2%; rheumatoid factor (RF)–negative polyarthritis, 15.6%; systemic arthritis, 12.2%; juvenile psoriatic arthritis, 5.2%; undifferentiated arthritis, 2.8%; and RF-positive polyarthritis, 2.2%. JIA-associated uveitis was observed in 6.8% of patients at a mean (Standard Deviation, SD) age of 9.1 (3.8) years over a mean JIA disease duration of 4 (1.9) years. Uveitis developed after joint disease, with a mean (SD) duration of 1.8 (1.9) years. Patients with oJIA had the highest rate of uveitis (12.9%) followed by patients with ERA (5.2%) and polyarticular RF-negative disease (3.8%). Compared with persistent oJIA, the extended oJIA category had a > 3-fold higher risk of uveitis (11.3% vs 27.7%; odds ratio, 3.38 [95% Confidence Interval, 1.09–10.4]). The most frequently administered drug after development of uveitis was tumor necrosis factor–alpha inhibitors (38.2%). Five patients (14.7%) had uveitis-related complications that required surgical intervention.

**Conclusions:**

Turkish pediatric patients with JIA experience a lower frequency of oJIA and higher frequency of ERA than their white European counterparts; the occurrence of uveitis is also somewhat lower than expected. Geographic and ethnic factors may affect these differences and need further investigation.

## Background

Juvenile idiopathic arthritis (JIA), the most common pediatric rheumatologic disorder, is a heterogeneous, chronic, childhood arthritis that lasts for ≥6 weeks, with onset before age 16 years [1, 2]. The incidence and prevalence rates of JIA vary depending on the geographic region, ethnicity and methodology used for inclusion of patients. In several studies, incidence rates of 2 to 20 per 100,000 individuals and prevalence rates of 7 to 400 per 100,000 individuals have been reported [3, 4]. So far, no population-based data are available regarding the distribution of JIA categories according to International League of Associations for Rheumatology (ILAR) and prevalence rates of JIA-associated uveitis (JIAU) in Turkey.

Morbidities and disabilities are frequently associated with JIA, including extra-articular complications. Uveitis, which is defined as the inflammation of the iris, ciliary body and choroid, is the most common and severe extra-articular complication [5]. If it remains untreated, uveitis can lead to sight-threatining complications, such as cataracts, glaucoma or band keratopathy. Noninfectious uveitis in children accounts for 65 to 90% of all childhood uveitis [6, 7]. JIAU, which occurs in 3 to 30% of children with JIA, has been reported to be the most frequent cause of chronic intraocular inflammation among children. The uveitis in JIA is frequently characterized by bilateral, nongranulomatous, chronic, relapsing and inflammatory episodes, most of which are asymptomatic [5].

There is need for an in-depth understanding of the influence of ethnicity and geographic factors on the distribution of JIA and the risk of developing JIAU to help drive future treatment strategies. Only limited data are available on distributions of JIA categories and the frequency of JIAU in Turkey.

The primary objective of this study was to provide up-to-date information regarding the frequency rate of uveitis in patients with JIA in Turkey. ﻿Secondary objectives included evaluation of the distribution of JIA categories according to the ILAR criteria, comparison of the frequency of uveitis and the uveitis-related complications between different categories of JIA and documentation of comorbidities.

## Materials and methods

### Study design, participants and randomization

This study was a national, noninterventional, multicenter, retrospective study conducted in four pediatric rheumatology clinics across three cities in Turkey.

Before study initiation, a randomization table was prepared that included 125 random numbers between 1 and 253. In each clinic, patients diagnosed with JIA between 01 January 2010 and 01 January 2017 were identified, and consecutive numbers were assigned to the eligible patients retrospectively, starting with the patient with the most recent date of diagnosis. Using this table, 125 patients were randomly assigned from all eligible patients at each center. Patient data were initially anonymized and de-identified by the entering physicians and then documented within a mutual electronic database.

This study was conducted in compliance with the Helsinki Declaration as well as local laws and regulations. Because of the retrospective nature of the study, obtaining written informed consent from the patients was not required. Ethics committee approval (date/no.: 03 October 2017/D-01) was obtained from the Istanbul University-Cerrahpasa, Ethics Committee of Clinical Trials.

### Enrollment process

Inclusion criteria included male and female patients ≤16 years of age diagnosed with JIA between 01 January 2010 and 01 January 2017, based on the 2001 revised ILAR criteria. Patients with incomplete medical data were excluded.

### Data collection

All of the patient data in this study were retrieved primarily from paper-based patient files. Complete medical charts, including demographics, diagnoses according to ILAR, laboratory test results, medications, were checked for any erroneous records by the entering physicians (all are pediatric rheumatologists), who were responsible from the transfer of these paper-based data into an electronic database.

The following items were documented during the collection of data from the patient records: patient demographics (age, sex, date of first and last follow-up visits, date of disease onset and JIA diagnosis), disease duration, ILAR category, laboratory parameters (C-reactive protein [CRP] and/or erythrocyte sedimentation rate [ESR], antinuclear antibody [ANA], rheumatoid factor [RF], human leukocyte antigen B27 [HLA-B27]) and comorbidities. Positive RF and ANA tests (with a titer ≥1/160 by immunofluorescence) were defined as presence of them on at least two occasions at least 3 months apart. If uveitis was noted, the medical charts were further reviewed for the following data: date of uveitis diagnosis; affected eye(s); localization and laterality of uveitis using the Standardization of Uveitis Nomenclature if available [8]; cumulative flare score; number of uveitic episodes both in the whole study period after disease onset and in the last 12 months prior to last visit; characteristics of these flares within the past 12 months (visual acuity, increased cell number in anterior chamber, vitreous haze). A 2-step or more increase in the degree of inflammation based on anterior chamber flare, anterior chamber cell score or vitreous haze score was defined as uveitic flare [8]. Laboratory parameters including CRP and/or ESR (if available), prescribed medications after development of uveitis and complications of uveitis requiring surgery were also recorded.

### Reimbursement of cost for bDMARDs in JIA and uveitis

Although patients have an unlimited access to health care in Turkey, certain criteria need to be met before the cost of treatment with biologic disease-modifying antirheumatic drugs (bDMARDs) for JIA and uveitis can be reimbursed. First, costs for biologic agents, including etanercept, adalimumab, tocilizumab and abatacept, are reimbursed only if American College of Rheumatology Pediatric 30 response has not been achieved after ≥3 months’ treatment with a conventional synthetic disease-modifying antirheumatic drug (csDMARD). Second, in systemic JIA (sJIA), tocilizumab and anakinra are reimbursed only if nonsteroidal anti-inflammatory drugs and systemic corticosteroids fail to induce remission after 3 months of therapy. Then, if remission is not achieved after ≥3 months of tocilizumab or anakinra therapy, canakinumab will be reimbursed. Third, patients with noninfectious uveitis who show no improvement after ≥3 months of csDMARD, qualify for reimbursement for adalimumab.

### Statistical considerations

Because of the exploratory study design and nonconfirmatory objectives, the sample size was based on practical considerations rather than on statistical power calculation. Statistical tests were performed using SPSS Statistics, version 21.0 (IBM Corp., Armonk, NY). Descriptive statistical analysis was conducted to summarize the study data. Summary statistics for continuous variables were given as mean, standard deviation; tabulations of categorical variables were presented in all possible categories and were displayed as the number of observations per category as well as percentages, where applicable. Missing values were not imputed via any assumptions, and statistical analyses were conducted with all available data. Number and percentage of patients per JIA category according to ILAR criteria and clinical/demographic categories were presented.

## Results

### Demographic characteristics and distribution of JIA categories

A total of 500 patients were recruited from four pediatric rheumatology referral centers, with 125 randomly selected from each clinic. Male patients constituted 42.2% of the study population (*n* = 211). Patients were aged between 9 months and 16 years, with a mean (Standard Deviation, SD) age of 8.8 (4.7) years at diagnosis, (Table 1). The most common JIA category among patients in this study, as per ILAR criteria, was oligoarticular JIA (oJIA; *n* = 194 [38.8%]) followed by enthesitis-related arthritis (ERA; *n* = 116 [23.2%]). Within the oJIA category, 90.7% (*n* = 176) were categorized as persistent oJIA (poJIA) and 9.3% (*n* = 18) as extended oJIA (eoJIA). Female patients made up a higher percentage of those with eoJIA (*n* = 14/18, 77.7%) than poJIA (*n* = 116/176, 65.9%).

**Table 1:** Demographic and clinical characteristics of the participants in the JUPITER cohort

	Assessed patients, n	Total cohort	ERA	oJIA	Poly JIA, RF–	Poly JIA, RF+	JPsA	sJIA	uJIA
All subjects, n (%)	–	500	116 (23.2)	194 (38.8)	78 (15.6)	11 (2.2)	26 (5.2)	61 (12.2)	14 (2.8)
Sex, female, n (%)	500	289 (57.8)	31 (26.7)	130 (67.0)	62 (79.5)	9 (81.8)	15 (57.7)	30 (49.2)	12 (85.7)
Age at JIA diagnosis, years, mean ± SD	500	8.8 ± 4.7	12.1 ± 2.9	6.5 ± 4.3	8.6 ± 4.5	11.6 ± 4.6	10.5 ± 3.4	7.5 ± 4.7	11.8 ± 3.5
Disease duration, years, mean ± SD	500	4.0 ± 1.9	3.6 ± 1.6	4.2 ± 2.0	4.6 ± 2.1	5.0 ± 2.3	3.2 ± 1.8	4.4 ± 2.9	2.4 ± 1.0
Comorbidities, n (%)	500	102^a^	20 (17.3)	28 (14.4)	13 (16.7)	0 (0)	15 (57.7)	14 (23.0)	2 (14.3)
FMF		63 (12.6)	19 (16.4)	26 (13.4)	6 (7.7)	0 (0)	0 (0)	10 (16.4)	2 (14.3)
Osteoporosis		8 (%1.6)	0 (0)	2 (1.0)	5 (6.4)	0 (0)	0 (0)	1 (1.7)	0 (0)
Delayed puberty^c^		6 (%1.2)	1 (0.9)	0 (0.9)	2 (2.6)	0 (0)	0 (0)	3 (4.9)	0 (0)
ANA positivity, n (%^b^)	396	191 (48.2)	10 (15.9)	127 (68.3)	31 (40.8)	2 (25)	8 (38.1)	9 (23.1)	4 (33.3)
HLA-B27 positivity, n (%^b^)	168	56 (33.3)	47 (51.7)	2 (5.9)	–	–	4 (23.5)	–	3 (23.1)

*ANA* antinuclear antibody, *ERA* enthesitis-related arthritis, *FMF* familial Mediterranean fever, *HLA* human leucocyte antigen, *JIA* juvenile idiopathic arthritis, *JPsA* juvenile psoriatic arthritis, *oJIA* oligoarticular JIA, *Poly JIA* polyarticular JIA, *RF* rheumatoid factor, *sJIA* systemic JIA, *uJIA* undifferentiated JIA

^a^ Total number of comorbidities encountered in JIA cohort

^b^ The values denote the ANA or HLA-B27 positivity rates among the assessed patients of the respective JIA category

^c^ Lack of secondary sexual characteristics by age 13 years in girls and age 14 in boys

### Laboratory and comorbidity data

One-third of the assessed JIA patients were HLA-B27 positive (*n* = 56/168 [33.3%]), of which 84% (*n* = 47/56) were in the ERA category (Table 1). Fifty-two percent (*n* = 47/91 [52%]) of patients in the ERA group and 2% (*n* = 4/17 [2%]) in the juvenile psoriatic arthritis (JPsA) group were HLA-B27 positive. Almost half (48.2%) of the assessed children (*n* = 396) in this JUPITER cohort were ANA positive, 66.5% (*n* = 127/191) of whom were in the oJIA group. Of the 186 oJIA patients who were assessed for the presence of ANA, 68.3% (*n* = 127/186) were positive. The rate of ANA positivity was higher in the eoJIA subcategory (*n* = 16/18 [89%]) than in the poJIA subcategory (*n* = 111/168 [66%]).

Of the 487 patients assessed for CRP at the time of JIA diagnosis, 294 (60.4%) had abnormal CRP values. Elevated CRP levels at JIA diagnosis were most frequently noted in RF-positive disease (*n* = 11/11 [100%]), followed by the sJIA category (*n* = 53/60 [88%]), RF-negative disease (*n* = 53/73 [73%]), ERA (*n* = 65/113 [58%]) and oligoarthritis category (*n* = 99/191 [52%]; 61% in eoJIA vs 48% in poJIA). Patients in the JPsA group were the least likely to have an elevated CRP level (*n* = 11/25 [44%]) at the time of JIA diagnosis. Of the 32 patients assessed for CRP at the date of uveitis diagnosis, 50% (*n* = 16) had high levels of CRP. However, whether a joint disease was associated with the uveitis at this time was not documented during data collection.

**Fig. 1:**
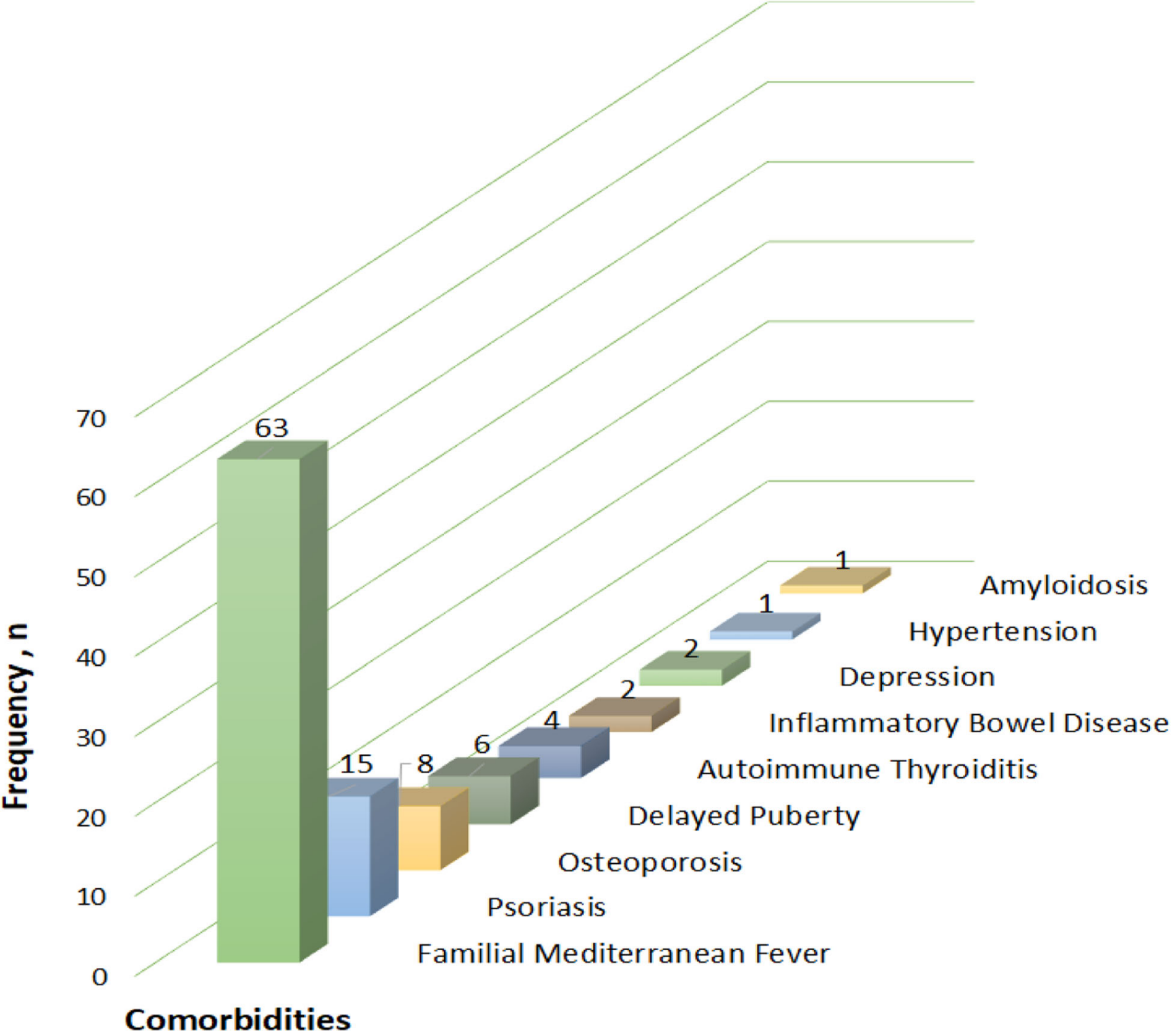
The comorbid conditions observed in the JUPITER cohort

A total of 102 comorbidities were reported in 500 patients (Fig. 1). The most frequently observed comorbidity was familial Mediterranean fever (FMF; *n* = 63 [12.6%]). Psoriasis was observed in more than half (57.7%) of the JPsA patients.

### Characteristics of patients who developed uveitis

Among 500 patients, uveitis developed in 34 (6.8%), whose ages ranged from 2.2 to 15.1 (mean [SD], 9.15 [3.8]) years at diagnosis. The percentage of female patients among those with uveitis was in keeping with the frequency among the total JIA population (58.8% vs 57.8%, respectively). Patients developed uveitis after the joint disease, with a mean (SD) duration of 1.8 (1.9) years. However, the diagnosis of uveitis was established before the diagnosis of JIA in 3 patients, all of whom were in the oJIA group. Uveitis occurred ≤2 years after the diagnosis of JIA in half of the JIAU group (*n* = 17/34). Nine patients out of 34 developed uveitis > 3 years: 3 within 4th year, 3 within 5th and 3 patients within 6th year (Table 2).

**Table 2:** Characteristics of patients with JIA-associated uveitis

Patient Characteristics	
Patients, n (%)	34 (6.8)
Sex, female, n (%)	20 (58.8)
Duration between onset of arthritis and uveitis, years, mean ± SD	1.8 ± 1.9
oJIA	1.9 ± 1.8
ERA	1.7 ± 2.1
Poly JIA, RF–	1.8 ± 3.0
Onset of uveitis after JIA diagnosis,^a^ years, n (%)	
0–1	13 (38.2)
1–2	4 (11.8)
2–3	5 (14.7)
> 3	9 (26.5)
Affected eye,^b^ n (%)	
Right only	5 (17.2)
Left only	8 (27.6)
Both	16 (55.2)
Localization of uveitis,^c^ n (%)	
Anterior	19 (76)
Intermediate	2 (8)
Posterior	2 (8)
Panuveitis	2 (8)
Categories of JIA/sex distribution, n (%) [female % within n]	
oJIA	25 (73.5) [64]
poJIA	20 (58.8) [65]
eoJIA	5 (14.7) [60]
ERA	6 (17.7) [9]
Poly JIA, RF–	3 (8.8) [100]
ANA positivity, n/N (%)	22/34 (64.7)
HLA-B27 positivity, n/N (%)	3/10 (30)
Systemic treatments initiated after the diagnosis of uveitis, n (%)	
Methotrexate	6 (17.6)
Azathioprine	4 (11.8)
Sulfasalazine	4 (11.8)
TNF-alpha inhibitors	13 (38.2)
Tocilizumab	3 (8.8)
Complications, n (%)	5 (14.7)
Cataract	4 (11.8)
Band keratopathy	1 (2.9)

*ANA* antinuclear antibody, *eoJIA* extended oJIA, *ERA* enthesitis-related arthritis, *HLA* human leucocyte antigen, *JIA* juvenile idiopathic arthritis, *oJIA* oligoarticular JIA, *poJIA* persistent oJIA, *Poly JIA* polyarticular JIA, *RF* rheumatoid factor, *TNF* tumor necrosis factor

^a^ The diagnosis of uveitis preceded the diagnosis of JIA in 3 patients

^b^ There were no data available for 5 patents

^c^ There were no data available for 9 patients

Overall, 64.7% of patients with uveitis were ANA-positive, with higher percentage of ANA-positivity detected in those with eoJIA (5/5) than those with poJIA (15/20).

﻿ Patients with oJIA had the highest rate of uveitis (*n* = 25/194 [12.9%]), followed by patients with ERA (*n* = 6/116 [5.2%]) and polyarticular RF-negative disease (*n* = 3/78 [3.8%]). However, it should be noted that the follow-up period for patients with ERA was shorter than for those in the oligoarticular and polyarticular groups (3.6 years vs 4.2 and 4.6 years, respectively). There was no uveitis in patients with sJIA, seropositive polyarticular JIA or JPsA.

Patients in the eoJIA group had a > 3-fold greater risk of uveitis than those in the poJIA group (5/18 [27.7%] vs 20/176 [11.3%]; *p* < 0.05; odds ratio, 3.38 [95% Confidence Interval,1.09–10.4]). The rate of uveitis was higher in oJIA patients who were ANA positive (*n* = 20/127 [15.7%]) than those who were ANA negative (*n* = 5/59 [8.5%]) (*p* < 0.05; odds ratio, 2.02 [95% Confidence Interval, 0.7–5.7]). However the frequency of uveitis did not differ between female and male patients in the oJIA group (*n* = 16/130 [12%] vs *n* = 9/64 [14%]; *p* > 0.05).

Among 34 patients with uveitis, eye involvement was present in both eyes, left eye and right eye in 16, 8 and 5 patients, respectively. Anterior uveitis was the most frequently detected anotomic form of uveitis in this study, occuring in 19 patients (76% of patients with available data about localization; followed by intermediate, posterior and panuveitis, each of which was seen in 2 patients.

Throughout the study period the median number of uveitic flares was 1.5 (range, 1–18) and the mean (SD) duration of uveitis was 3.2 (2.3) years. Seventeen of 34 patients (50%) had only one episode of uveitis. There has been no uveitic flare in 15 patients (44.1%) with JIAU in the past 12 months. Among the 19 patients who flared at least once over a 12-month period, 7 were excluded owing to missing data in ophthalmologic examinations. Focusing on the 12 patients with JIAU who had flared in the past 12 months and had no missing data, increased cell number in the anterior chamber was the most commonly noted finding during an episode of uveitis, occurring in 12 patients (100%), followed by worsening of visual acuity in 11 patients (91.7%) and vitreous haze in 4 patients (31.6%). The number of episodes ranged from one to six, with a median of one flare per year.

### Medications for uveitis and uveitis-related complications

Following diagnosis of uveitis, 14 of the 34 patients received csDMARDs and 16 received bDMARDs. There were no available data regarding systemic corticosteroids or topical therapy. The most frequently prescribed DMARD group after development of uveitis was tumor necrosis factor–alpha inhibitors (TNFi; 38.2%). Neither csDMARDs nor bDMARDs were initiated in four of the 34 patients with uveitis (Table 2).

Five of the 34 patients (14.7%) required surgery owing to uveitis-related complications. Cataract was detected in 3 patients with oJIA and in 1 patient with ERA. Band keratopathy and posterior synechia were detected in one patient with RF-negative polyarticular JIA.

## Discussion

In this retrospective and multicenter study of JIA in Turkey, category distribution differed from that in Europe and North America; the rate of ERA was higher, and the rate of oJIA was lower.

The frequency of JIA categories vary considerably throughout the world, possibly depending on ethnic differences as well as immunogenetic and enviromental factors. Globally, RF-positive polyarthritis is the least common category, and the most common category is oJIA [10]. In Europe, North America and Australia the most common JIA category is oJIA in virtually all pediatric reports, followed by RF-negative polyarthritis [9, 11–19], whereas in Asia, ERA and occasionally sJIA, representing 36 to 40% and 28 to 39% of all JIA categories respectively, appear to be predominate [20–24]. The reported frequency of oJIA has varied considerably, from 21 to 32% in Asia and Africa [20–26] to 36–39% in North America and Australia [9, 17–19] and to 42–55% in Europe [11–16]. A similar geographic variation exists in the distribution of poJIA and eoJIA, both of which are subcategories of oJIA. In our study, poJIA was observed approximately 10 times more frequently than eoJIA. This is, to our knowledge, the highest poJIA-to-eoJIA ratio to date. Likewise, a high ratio (9.2:1) has also been detected in a previous multicenter Turkish study [27], a proportion that has ranged from 1.3 to 6.5:1 in various studies from other countries [11–13, 16–18, 21–24]. Indeed, the frequency of poJIA in Turkey is similar to or even higher than that observed in Western Europe and North America [11–13, 17, 18]. In other words, the overall lower frequency of oJIA in our study is related to the lower rate of the eoJIA subcategory. In a Canadian study of a multiethnic JIA cohort by Saurenmann and colleagues, the higher frequency rates of oJIA in children of European versus non-European origin was thought to be mainly due to a higher frequency of eoJIA because the prevalence rates of poJIA were similar between the two ethnic groups [17]. The variations in the prevalence of JIA categories are believed to be determined by a complex interplay between genetic and geographic factors. The geographic position of Turkey between Asia and Europe and the presence of a notable genetic similarity with the East Asian and Southern European populations might be an explanation for why Turkish JIA patients are an average of two populations in terms of JIA category distribution [28].

The most common comorbidity in patients with JIA in this study was FMF (12.6%), which was considerably higher than the general prevalence of FMF in Turkey (1/1070) [29, 30]. Previously, higher carrier frequency of the FMF gene (MEFV) mutations were demonstrated in patients with JIA and sJIA [31, 32]. These findings suggest that MEFV variants may increase the susceptibility to JIA. However, the association between the MEFV gene and JIA is yet to be made clear.

Our study showed a lower rate of uveitis, which developed in 34 of 500 (6.8%) patients with JIA over a period of 4 years. The prevalance rates of uveitis in patients with JIA are highly variable throughout the world, ranging from 3.4 to 16% [12–16, 19, 21–24, 26, 27]. In the large multiracial Canadian cohort of patients with JIA, those of European descent had a 3- to 4-fold increased risk of developing uveitis compared with those of non-European origin [17].

The frequency of uveitis in any JIA cohort seems to correlate with the incidence of the oJIA category in that cohort and is rare in patients with RF-positive polyarticular JIA and sJIA [16]. Lower rates of uveitis have been reported in Asian countries where oJIA has been less commonly recognized [21–24]. Accordingly, higher rates of JIAU have been observed in Europe and North America, where oJIA predominates over other categories [12–14, 16, 18, 33]. As has been reported by other groups, we observed that patients with oJIA had the highest rate of uveitis, followed by those with ERA and RF-negative polyarticular JIA [18, 33]. A higher percentage of patients with eoJIA than patients with poJIA developed uveitis during the study period, as occurred in the study by Saurenmann and colleagues [33]. Intriguingly, a Canadian multiethnic JIA study demonstrated that children of European ancestry, were more likely to develop oJIA (particularly the eoJIA subcategory), have an early disease onset and a higher female-to-male ratio [17]. It is still unclear that whether the increased risk of developing uveitis in those of European ancestry could be attributable to ethnicity per se or to higher rates of oJIA or the eoJIA categories, female sex, ANA positivity or any combination of these in European JIA patients [34]. The present study demonstrates a lower incidence of JIAU, which possibly could be related to lower rates of oJIA, particularly the eoJIA subcategory and easy access to sytemic immunosuppressive drugs and biologic agents for joint disease. Uveitis occurred in approximately one-fourth of our patients > 3 years after diagnosis of JIA, which means a continued vigilance is warranted for eye involvement.

It is uncertain whether a temporal variability in the incidences of JIA and JIAU exists. There is a growing body of evidence that the occurrence of JIA is increasing, but the frequency of JIAU is decreasing [16, 33, 35, 36]. There is at least a 30 to 40% decrease in the prevalence of JIAU in our cohort when compared with two previous Turkish JIA cohorts [27, 37] with similar JIA category distributions and follow-up periods. A lower rate of uveitis was also observed in a single-center retrospective Turkish JIA cohort [38]. A recent Nordic population-based study confirmed diminished rates of JIAU, with a 50% decrease comparing to a previous Nordic JIA cohort with a corresponding duration of follow-up of approximately 20 years [12, 39].

Higher female-to-male ratios have been reported in oJIA patients with uveitis than those without uveitis [33]. However, we found a similar predominance of female patients in the oJIA group and oJIA plus uveitis group (67 and 64%, respectively). Consistent with this finding, a large German study failed to show female sex as an independent risk factor for the occurrence of JIAU [16].

Five patients required surgery for uveitis-related complications in our study. The patients whose diagnosis of uveitis precede the diagnosis of JIA, have more adverse outcomes in terms of uveitis-related complications [40]. In our study, this type of disease onset was present in 3 patients, 1 of whom developed cataract.

The introduction of biological medications has provided a very important new therapeutic option for the treatment of patients with JIA and JIAU who are resistant to csDMARDs. Adding biologic agents, specifically TNFi, is highly recommended in cases of methotrexate intolerance or inefficacy in JIAU [41, 42]. As in other developed countries, biologic agents including TNFi, interleukin-1 inhibitors and tocilizumab have been available for children with JIA and JIAU in Turkey since the early 2000s. The most frequently prescribed bDMARD group in our study cohort was TNFi (38.2%).

We are aware of the possible limitations associated with the retrospective design of this cohort study and that, because the data were collected from the perspective of pediatric rheumatologists, complications and characteristics of uveitic flares may have been underrepresented in this study. The possibility of a referral bias towards more patients with systemic and polyarticular JIA categories is another shortcoming of this study. On the other hand, avoidance from overrepresentation of any type of JIA category by the randomization of all patients diagnosed with JIA within a particular time period and the inclusion of patients from pediatric rheumatology referral clinics of the three most populated cities with sufficient national heterogeneity are the strengths of our study.

## Conclusions

In conclusion, the main differences of Turkish JIA patients from their European counterparts were lower frequency of oJIA but higher frequency of ERA and vice versa when compared to the Asian counterparts. And, the relative lower rate of oJIA in our JUPITER cohort is primarily attributable to the lower frequency of the eoJIA subcategory rather than the poJIA group. The frequency of uveitis was somewhat lower than expected and also lower than the rate in a previous Turkish JIA cohort. ﻿Our results support the hypothesis that incidence of JIAU has been decreasing over the years. The effect of geographic and ethnic factors, increasing rates of bDMARD usage on these findings should be investigated in larger cohorts. The rate of uveitis was higher in patients with oJIA, particularly in those who were ANA positive or in eoJIA subcategory. TNFi are the most frequently administered DMARDs by Turkish pediatric rheumatologists (and ophthalmologists) in treating JIAU.

## Data Availability

The datasets used and analysed during the current study are available from the corresponding author on reasonable request. No information (including demographic data, initials, date of birth), that would enable identification of any patient, were recorded or requested to maintain subject confidentiality; only the patient ages were collected. To protect a patient’s identity, a unique number was assigned to each patient. For each site, an account was provided for the electronic database. The participating site was asked to fill out the electronic form prepared in English. The format of this electronic record was decided before initial submission to the Ethics Committee. Accordingly, each center documented patient data in this database. Examinations, diagnostic measures, findings and observations routinely performed in patients included in this retrospective study were entered by the researcher into this electronic database provided by AbbVie, according to the research plan.

## Abbreviations

ANA: Antinuclear antibody
bDMARDs: Biologic disease-modifying antirheumatic drug
CRP: C-reactive protein
csDMARD: Conventional synthetic disease-modifying antirheumatic drug
eoJIA: Extended oJIA
ERA: Enthesitis-related arthritis
ESR: Erythrocyte sedimentation rate
FMF: Familial Mediterranean fever
HLA-B27: Human leukocyte antigen B27
ILAR: International League of Associations for Rheumatology
JIA: Juvenile idiopathic arthritis
JIAU: JIA-associated uveitis
JPsA: Juvenile psoriatic arthritis
JUPITER: Frequency of **J**uvenile Idiopathic Arthritis (JIA) subgroups and JIA-associated **U**veitis among JIA patients admitted to referral **P**ediatric rheumatology clinics **I**n **T**urk**E**y: A **R**etrospective Study
MEFV: FMF gene
oJIA: Oligoarticular JIA
poJIA: Persistent oJIA
RF: Rheumatoid factor
SD: Standard Deviation
sJIA: Systemic JIA
TNFi: Tumor necrosis factor–alpha inhibitors
